# Fucoxanthin administration delays occurrence of tumors in xenograft mice by colonospheres, with an anti-tumor predictor of glycine

**DOI:** 10.3164/jcbn.18-45

**Published:** 2018-07-25

**Authors:** Masaru Terasaki, Naoya Matsumoto, Ryuichi Hashimoto, Tetsuya Endo, Hayato Maeda, Junichi Hamada, Kazumi Osada, Kazuo Miyashita, Michihiro Mutoh

**Affiliations:** 1 School of Pharmaceutical Sciences, Health Sciences University of Hokkaido, 1757 Kanazawa, Ishikari-Tobetsu, Hokkaido 061-0293, Japan; 2 Cancer Prevention Laboratories, Health Sciences University of Hokkaido, 1757 Kanazawa, Ishikari-Tobetsu, Hokkaido 061-0293, Japan; 3 Faculty of Agriculture and Life Science, Hirosaki University, 3 Bunkyo-cho, Hirosaki, Aomori 036-8561, Japan; 4 School of Nursing and Social Services, Health Sciences University of Hokkaido, 1757 Kanazawa, Ishikari-Tobetsu, Hokkaido 061-0293, Japan; 5 School of Dentistry, Health Sciences University of Hokkaido, 1757 Kanazawa, Ishikari-Tobetsu, Hokkaido 061-0293, Japan; 6 Laboratory of Biofunctional Material Chemistry, Division of Marine Bioscience, Graduate School of Fisheries Sciences, Hokkaido University, Hakodate, Hokkaido 041-8611, Japan; 7 Epidemiology and Preventions Group, Center for Public Health Sciences, National Cancer Center, 5-1-1 Tsukiji, Chuo-ku, Tokyo 104-0045, Japan

**Keywords:** fucoxanthin, carotenoid, colorectal cancer stem cell, metabolite, xenograft mice

## Abstract

Fucoxanthin and its major metabolite, fucoxanthinol, have potent anti-cancer properties in carcinogenic model mice and against cancer cells. Evidence has accumulated regarding the diagnostic potential of biological metabolites as invasive and non-invasive obtainable approaches. We recently demonstrated that glycine was an effective predictor of the suppression of sphere formation and epithelial mesenchymal transition by fucoxanthinol in human colorectal cancer stem-like spheroids (colonospheres) under normoxia and hypoxia. In the present study, we investigated the suppressive effect of fucoxanthin on tumorigenesis derived from colonospheres in xenograft mice, and the alteration on the metabolite profiles of mouse tumors by fucoxanthin was evaluated. Fucoxanthin administration at 2.5 mg/kg body weight (p.o.) for 4 weeks significantly inhibited the incidence of tumors by inoculation of colonospheres suspension in BALB/c *nu*/*nu* mice compared with control mice, but not tumor sizes. In addition, fucoxanthin down-regulated tumor Cyclin D1 expression by 0.7-fold of that observed in the tumors of the control mice. Moreover, the tumor glycine level in the xenograft mice was decreased by fucoxanthin administration to 0.5-fold. These results imply the possibility of tumor metabolites as a prediction marker of tumorigenicity derived from colorectal cancer stem cells in mice.

## Introduction

**Fig. 1:**
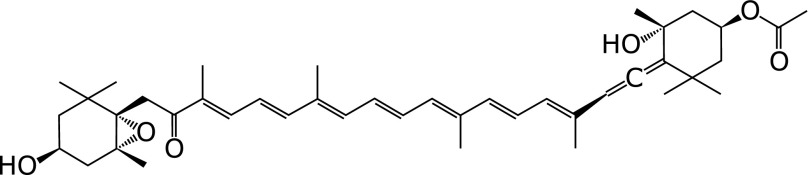
Fucoxanthin. MW: 658.91.

Fucoxanthin (Fx), a nonprovitamin A carotenoid, is a well known major xanthophyll contained in edible brown algae and has shown cancer chemopreventive effects in several cancer mice models (Fig. 1).^(1,2)^ Toxicity studies (up to 2,000 mg/kg body weight) revealed that Fx could be safe, i.e., not showing adverse effects.^(3,4)^ Fucoxanthinol (FxOH), a deacetylated form of Fx, is the main metabolite observed in human and mouse blood after Fx or brown algae ingestion.^(5–9)^ It is reported that Fx and FxOH powerfully inhibit cell growth in primary cultured colorectal cancer (CRC) cells,^(10)^ and in various cultured cancer cell lines.^(11–17)^ However, the anti-cancer mechanisms of Fx and FxOH have only partially been determined.

CRC is one of the 4th common cause of deaths for neoplasia in the world, and therefore it is urgent to prevent its development and progression.^(18)^ CRC stem cells (CCSCs), which occupy a minor subpopulation of CRC cells and possess the potential of self-renewal, differentiation, sphere formation and tumorigenicity, are thought to play a central role in carcinogenesis.^(19,20)^ Recently, the three-dimensional tumor cells formed from CRC cells, called colonospheres (Csps), have been considered to be a representative CCSC model phenotype because they contain a high abundance of CCSCs and possess sphere reconstruction and tumorigenic capacities.^(19–21)^

Low molecular weight metabolites, such as amino and carboxylic acids, not only play an important role in regulating energy metabolism but also indicating the condition of many diseases, including cancer. To date, several metabolite index candidates in CRC patients and animals have been demonstrated in invasive and non-invasive cancers.^(22–24)^ We have recently reported that intracellular glycine and succinic acid indices correlated with suppression of sphere formation due to FxOH treatment in Csps.^(25,26)^ Moreover, we have recently demonstrated that FxOH induces apoptosis in Csps and inhibits their tumorigenicity in a xenograft mouse model,^(27)^ while previous studies have not explored differences of biological metabolites in tumor-bearing mice with FxOH or Fx administration.

In the present study, we investigated the suppressive effect of Fx on tumorigenesis derived from cancer stem-like Csps in xenograft mice. Further, we evaluated the changes caused by Fx on the metabolite profiles of mouse tumors, and imply the possibility of tumor metabolites as a prediction marker of tumorigenicity derived from colorectal cancer stem cells in mice.

## Materials and Methods

### Chemicals and cell culture

Fx-WSP0.1KW, a Fx powder composed of 0.1 weight% of Fx and slight carbohydrate, lipids and sodium per 100 g dry weight, was kindly donated by Oryza Oil & Fat Chemical Co. Ltd. (Aichi, Japan). Human colon adenocarcinoma cell line HT-29 was purchased from American Type Culture Collection (Rockville, MD). These cells were cultured in Dulbecco’s modified Eagle’s medium (DMEM) supplemented with 10% heat-inactivated fetal bovine serum, 4 mM l-glutamine, 40,000 U/L penicillin, and 40 mg/L streptomycin. Epidermal growth factor (EGF), and basic fibroblast growth factor (bFGF) were purchased from Wako Pure Chemicals (Osaka, Japan). DMEM/F12 and B27 supplement were obtained from Life Technologies (Gaithersburg, MD) and Miltenyi Biotec (Auburn, CA), respectively. The cells were cultured at 37°C in a humidified atmosphere of 95% air and 5% CO_2_. All other chemicals and solvents were of analytical grade.

### Culture and colonosphere formation

Adherent HT-29 parent cells were trypsinized from culture plates, washed with PBS twice, suspended in stem cell medium (SCM) composed of DMEM/F12 medium, 20 ng/ml EGF, 10 ng/ml of bFGF, 0.2% of B27 and antibiotic-antimycotic agent, plated at a density of 3 × 10^4^ cells/ml SCM into 24-wells of an ultra-low attachment plate (Corning, NY) and incubated for 2 days at 37°C in a humidified atmosphere containing 5% CO_2_. All experiments with Csps described below were performed using Csps grown for 2 days.

### Xenograft tumor experiments

The experimental scheme is shown in Fig. 3A. In brief, twenty male BALB/c *nu*/*nu* mice (five weeks old) were purchased from Japan SLC Inc. (Shizuoka, Japan). Five mice each were housed in plastic cages with sterilized softwood chips as bedding in a humidity- and temperature-controlled animal room on a 12 h light/dark cycle. Solid food (Grade: MF, Oriental Yeast Co. Ltd., Tokyo, Japan) and water were fed *ad libitum*. After a week of acclimation, the single cell suspension dissociated from the Csps was subcutaneously inoculated onto the right femoral region of all BALB/c *nu*/*nu* mice at 1 × 10^5^ cells/100 µl 0.1% BSA/PBS using a 25.0-gauge 1 ml disposable syringe. The animals were observed daily for clinical signs and mortality. The estimated tumor volume was expressed as the formula of a (mm) × b^2^ (mm)/2 (a, long range: b, short range). Fx powder was suspended in water at 0.01 w/v% and administered per mouse at 2.5 mg Fx/kg body weight (0.01 w/v% in water) using a stomach needle every 2 or 3 days for 3 weeks. The control group was given the equivalent volume of water only for each mouse. Tumor size and body weight were measured at the time of Fx administration. The no xenograft BALB/c *nu*/*nu* mice with and without Fx administration were prepared as control groups. The mice were injected with urethane (1.0 g/kg, i.p.) and pilocarpine (5 mg/kg, s.c.) 1 h before sacrifice, and then saliva (<500 µl) was collected for 30 min, and then a blood sample and the subcutaneous tumor were taken immediately. The experiments were performed according to the “Guidelines for Animal Experiments in the Health Sciences University of Hokkaido” that were approved by the Institutional Ethics Review Committee for Animal Experimentation in the Health Sciences University of Hokkaido.

### Western blot

β-Catenin and Cyclin D_1_ antibodies were purchased from Cell Signaling Technology (Danvers, MA). β-Actin, E-Cadherin, LGR5 and Vimentin antibodies were from GeneTex (Irvine, CA). CD44 and MMP-9 antibodies were from Thermo Fisher Scientific (Waltham, MA). EpCAM antibody was from EXBIO (Prague, Czech). Csps and tumor tissues were harvested, washed twice with PBS and then lysed in a lysis buffer for whole cell lysates. Fifty micrograms of whole cell proteins were separated on SDS-polyacrylamide minigels. The gels were then electroblotted onto a PVDF membrane. The PVDF was incubated in 1% BSA blocking buffer at room temperature and was probed with each of the primary antibodies in the blocking buffer, following the manufacturer’s instructions, overnight at 4°C. The membranes were washed and incubated with HRP-conjugated anti-mouse or anti-rabbit secondary antibodies. The membranes were washed and subsequently subjected to chemiluminescence reagents.

### GC-MS analysis

The tumor tissue was re-suspended in 50 µl of cold PBS, to which 0.05 µg of 2-isopropylmalic acid was added as an internal standard, and then the suspensions were disrupted by sonication for 5 s on ice. Total protein contents were determined using the Bradford method with 1 µl of each suspension. One hundred microliter aliquot of collected saliva and serum was divided, 0.05 µg of 2-isopropylmalic acid was added. Then the measured amount of each sample was extracted with 0.5 ml of CH_3_OH/CHCl_3_/DW (2.5:1:1, v/v/v), centrifuged at 16,000 *g* for 5 min and the upper phase was washed with 0.5 ml of DW. The upper extracts obtained were evaporated to dryness. The residues were derivatized by methoxyamine hydrochloride and *N*-methyl-*N*(trimethylsilyl)-trifluoroacetate. GC-MS analyses were performed using a GCMS-QP5000 instrument (Shimadzu, Kyoto, Japan) fitted with a Rxi-5ms column (30 m × 0.25 mm i.d., film thickness 0.25 µm; RESTEK Co. Ltd., GmbH, Bad Homburg, Germany). Helium was used as the carrier gas at 0.5 ml/min (15.7 kPa) and samples (1 µl) were injected in split-ratio mode (split ratio, 33%). Column temperature was initially 80°C for 2 min and increased at a heating rate of 330°C at 4°C/min, and then held at 330°C for 8 min. The temperature of the ion source and interface were 230 and 250°C, respectively. Identification was confirmed by mass spectra and retention times of authentic standards and secured by comparison of the spectra of the single components with those stored in the acquisition system library. Metabolite contents were expressed in pmol metabolite per mg tumor protein content, 100 µl-saliva or -serum.

### Statistical analysis

All the results are expressed as mean ± SE values. Significant differences between the means of two groups were determined by Chi-squared test for tumor incidence in xenograft mice and by Wilcoxon rank sum test for other all comparisons, and the differences were considered statistically significant when *p*<0.05 (*).

## Results

### Stemness of colonospheres

**Fig. 2:**
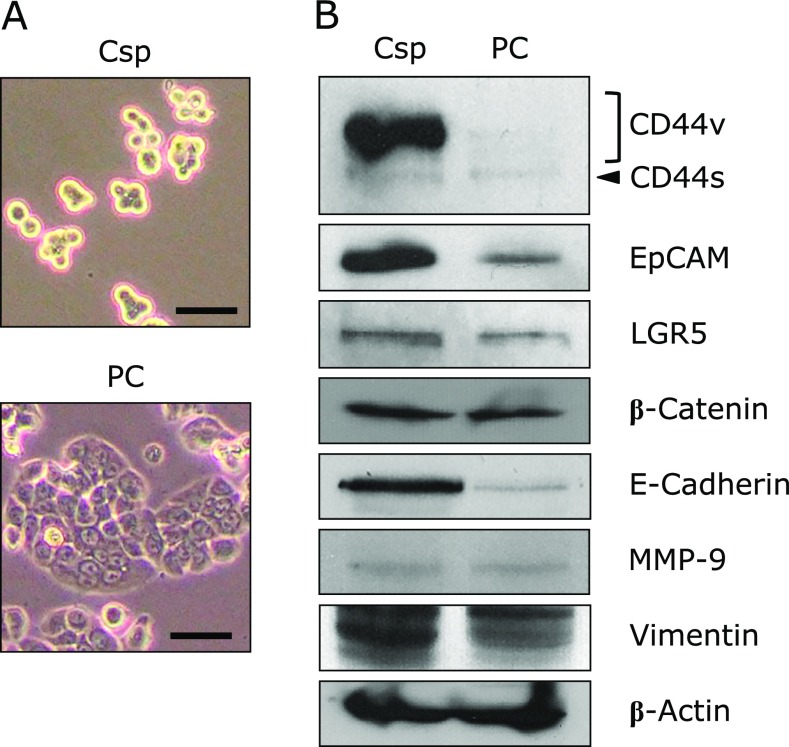
Marker proteins related to colorectal cancer stem cells and the epithelial-mesenchymal transition of colonospheres formed from HT-29 parent cells. Colonospheres (Csps) were prepared with stem cell medium for 2 days. (A) Images of Csps and the parent cells (PCs) of HT-29 cells by phase-contrast microscopy. Bar, 100 µm. (B) Csps and PCs were collected, and their protein levels were determined by western blotting (details are described in Materials and methods section).

To evaluate the stemness characteristics of cancer stem-like Csps just before the inoculation to BALB/c *nu/nu* mice, the protein profiles of the Csps formed from HT-29 cells were investigated and compared with the parent cells (PCs) (Fig. 2A). CCSC surface markers such as CD44 variant forms (CD44v), EpCAM and LGR5 were overexpressed in Csps compared with PCs. The expression of β-catenin, a key molecule for Wnt/β-catenin pathway, did not change between the Csps and PCs. Among three essential regulators of the EMT phenotype, E-Cadherin and Vimentin increased in Csps compared with PCs. MMP-9 was not altered between the two cell types (Fig. 2B).

### Effect on xenograft mice by Fx administration

**Fig. 3:**
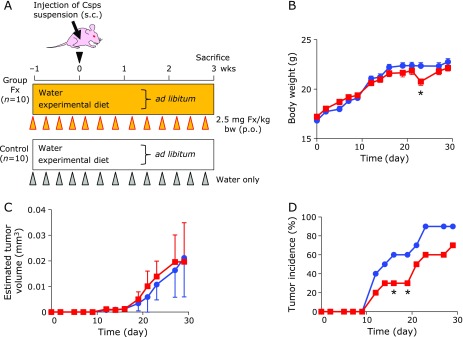
Body weight, tumor incidence and size of colonospheres-xenografted mice with or without fucoxanthin (Fx) administration. Colonospheres (Csps) were developed with stem cell medium for 2 days. (A) The single-cell suspension dissociated from Csps was injected subcutaneously on the back of the BALB/c-nu/nu mice at 3 × 10^4^ cells/100 µl (0.1% BSA/PBS). Fx powder was dissolved in water (0.01 w/v%) and was gavaged at 2.5 mg Fx/kg body weight (0.01 w/v% in water) using a stomach needle every 2–3 days from 1 week before Csps inoculation to 4 weeks after. The control group was given an equivalent volume (µl) of water alone. Significant differences in body weight (B) and estimated tumor size (C) were performed by Wilcoxon rank sum test (vs control). Means ± SE (*n* = 10). *p<0.05. Significant differences in tumor incidence (D) are evaluated by Chi-squared test. *p<0.05. Red line: Fx-treated group; Blue line: control group.

Fx in water was administered at 2.5 mg Fx/kg body weight (0.01 w/v% in water)**by gavage every 2–3 days for 4 weeks, i.e., for 3 weeks from when the Csps suspension was subcutaneously inoculated (Fig. 3A). Administration of Fx to BALB/c *nu/nu* mice for 4 weeks did not affect food intake and clinical signs throughout the experimental period. The weights of the liver and right kidney in the mice tended to decrease by administration of Fx in comparison with the control mice at the sacrifice time point, however, there was no significant difference in the left kidney, spleen and tumor weights (Table 1). Little difference in body weight was observed between the Fx-treated mice and the control mice during most of the experimental periods, except for day 23 of unknown etiology.

The tumor volumes were approximately the same between both mouse groups until the end of the experiment (Fig. 3C). The tumor incidence of the Fx group (30%) showed a significant delay at 16 and 19 days compared with the control mice (60%) (*p<0.05) (Fig. 3D).

**Fig. 4:**
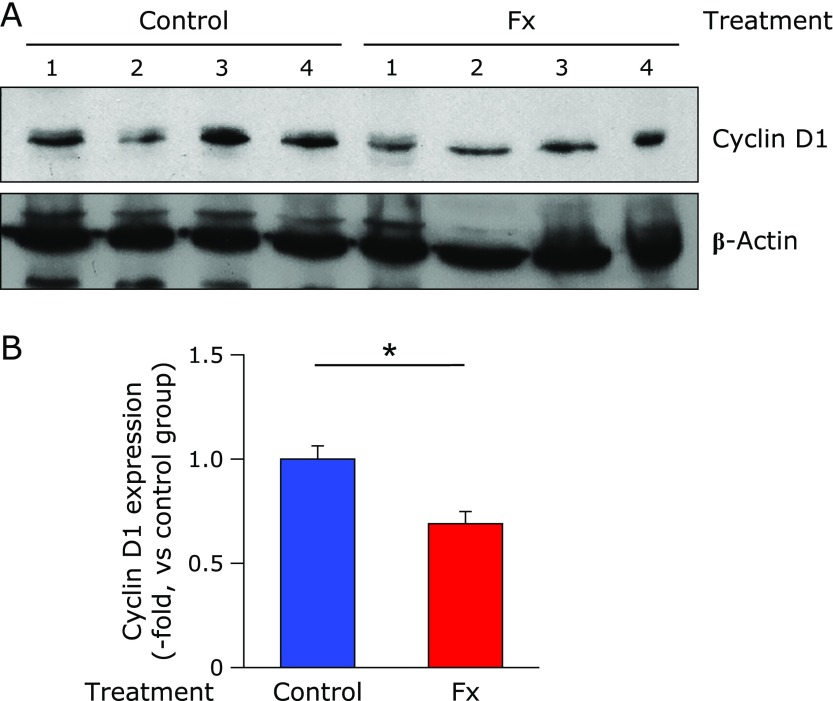
Cyclin D1 protein expression in tumor tissues from colonosphere-xenografted mice with or without fucoxanthin (Fx) treatment. BALB/c-nu/nu mice inoculated with Csps suspension were treated with or without Fx for 3 weeks. (A) The tumors at the end of the experiment were collected and subjected to western blot. The amount of the protein was measured, and the same amount of protein was loaded. (B) The value of each band was normalized to that of the β-actin band density from the image. Values are means ± SE (control group, *n* = 9; Fx-treated group, *n* = 5). *p<0.05 (Wilcoxon rank sum test, vs control).

To evaluate the protein expression for a key regulator molecule of cell cycle, Cyclin D1, we carried out western blot analysis. Fx administration significantly down-regulated Cyclin D1 expression to 0.7-fold of the control mice level (*p<0.05) (Fig. 4A and B).

### Change of metabolite profile in tumor tissue, saliva and serum of xenograft mice by Fx administration

**Fig. 5:**
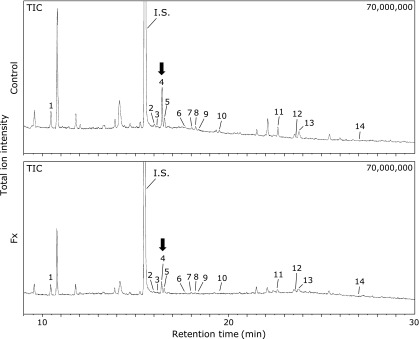
GC-MS total ion chromatograms of tumor metabolites in colonosphere-xenografted mice with or without fucoxanthin (Fx) treatment. BALB/c-nu/nu mice inoculated with Csps suspension were treated with or without Fx for 3 weeks. The tumor at the end of the experiment was collected, the metabolites were extracted and then analyzed by GC-MS. Peak numbers are indicated as follows: 1, butyric acid; 2, isoleucine; 3, proline; 4, glycine (arrow); 5, succinic acid; 6, fumaric acid; 7, pelargonic acid; 8, alanine; 9, serine; 10, threonine; 11, malic acid; 12, aspartic acid; 13, γ-aminobutyric acid; 14, glutamic acid. The information of tumor metabolites corresponding to each peak number is shown in Table 2. GC-MS conditions are given in Materials and Methods section.

Tumor metabolite profiles in Fx-treated mice were investigated by GC-MS (Fig. 5). The quantitative data of the metabolites analyzed in the xenograft mice treated with or without Fx with comparative data of saliva and serum metabolites are shown in Table 2. Glycine was significantly decreased in Fx-treated mice relative to the untreated-control mice as follows: glycine: Fx 6.7 ± 1.1 (0.5-fold vs control 13.9 ± 2.8) at pmol metabolite/mg tumor protein content. In comparison, salivary glycine, glutamic acid and succinic acid were significantly increased in Fx-treated mice compared with the untreated-control mice as follows: glycine: Fx 272.2 ± 36.5 (2.2-fold vs control 121.7 ± 29.8); glutamic acid: Fx 51.6 ± 10.9 (2.9-folds vs control 17.9 ± 2.4); succinic acid: Fx 52.1 ± 4.3 (2.0-folds vs control 25.7 ± 3.0) at pmol metabolite/100 µl saliva. Serum metabolites were not changed between the two mouse groups (Table 2).

## Discussion

We suggested that Fx administration suppressed the development of subcutaneous tumors, engrafted cancer stem-like Csps, along with Cyclin D1 suppression in BALB/c *nu*/*nu* mice. Glycine was revealed to be a prognostic biomarker of tumors in the xenograft mice model. This is the first report demonstrating tumor indicators in xenograft mice with Fx treatment.

We first investigated the expression of stemness-related proteins (cell surface markers and EMT phenotype) in HT-29 Csps before inoculation of the cells into BALB/c *nu*/*nu* mice (Fig. 2). The expression levels of CD44v, EpCAM, LGR5, E-Cadherin and Vimentin in HT-29 Csps increased in comparison with PCs. The anti-CD44 antibody we used in this study could not be able to divide further variant CD44s that we could not investigate in detail regarding variant CD44s this time. β-Catenin and MMP-9 did not show significant differences between the Csps and PCs. It is considered that the transformation of epithelial cancer cells to the EMT phenotype is due to decreased E-Cadherin and increased Vimentin and MMP-9 expressions. In the present study, however, the parallel up-regulations of E-Cadherin and Vimentin were observed and no alteration of MMP-9 in the Csps compared to the PCs. We used HT-29 Csps for the xenograft experiment because we believe that cell surface markers might be a more specific factor of the stemness than the EMT phenotype.

We next aimed to clarify the suppressive potential of Fx on tumorigenicity in BALB/c *nu*/*nu* mice. Intragastric administration of Fx (0.01 w/v% in water) at 2.5 mg/kg body weight every 2 or 3 days for 4 weeks tended to delay the tumor incidence compared to that of the control mice (Fig. 3D). Moreover, Fx down-regulated protein expression of an essential regulator for the cell cycle, Cyclin D1, in the xenografted tumor tissues (Fig. 4). It has been reported that free drinking of Fx in 0.01 w/v% water for 7 weeks inhibited the number of 1,2-dimethylhydrazine-induced colon aberrant crypt foci (ACF) in male B6C3F1 mouse.^(28)^ Similarly, in azoxymethane-induced colon ACF in ddY mice, Fx administration at a dose of 0.005 or 0.01 w/v% water for 4 weeks resulted in a decrease of ACF development.^(29)^ Our present data and those two prior results suggest that Fx administration at several mg/kg body weight every day or few days in drinking water exerts a chemopreventive effect on mouse carcinogenic models. In addition, we also demonstrated that oral administration of 5 mg/kg body weight of FxOH oil, a representative intestinal metabolite of Fx, to an immunodeficient NOD/SCID mice every 3 or 4 days resulted in a temporary reduction of tumorigenicity.^(27)^ Of note, the Fx in water has a slight caloric content such as carbohydrate, lipids and sodium derived from Fx-WSP0.1KW as follows: carbohydrate, 98.8 mg; lipids, 1.0 mg; sodium, 4.4 µg per ml. Hence, it is not strict control water. However, we assumed that the slight caloric content in the drinking water would not affect tumor incidence because the forced ingestion ratio of these three components into the mice was calibrated as less than 3 wt% against those obtained from daily MF intake.

Our previous studies demonstrated that glycine and succinic acid under normoxia or glycine under hypoxia were metabolite indicators correlated with suppression of sphere formation and epithelial mesenchymal transition due to FxOH treatment in Csps.^(25,26)^ In the present study, the tumor glycine content in the xenograft mice was significantly decreased by Fx administration to 0.5-fold (Fig. 5 and Table 2). Therefore, Fx administration may have occurred with a similar mechanism to that of FxOH-treated Csps against the tumors of xenograft mice by Csps. In addition, salivary glycine, glutamic acid and succinic acid may serve as indicators for the biological condition in xenograft-mice by Fx administration (Table 2). The concentration of these indicators all increased more than two-fold in the saliva by Fx administration for 4 weeks. Although the reason why salivary metabolome profiles are altered in cancer patients is not clear yet, many salivary amino acids and their derivatives, such as alanine, glutamic acid, glycine, leucine, valine, taurine, pipecolic acid and ornithine could be useful as surrogate markers to discriminate human cancers, such as breast, oral and pancreatic cancers. Many salivary metabolites of both patients with breast and pancreatic cancers are shown to be increased in comparison with healthy controls.^(30)^ As is assumed, most salivary metabolites tend to increase in patients with oral cancer.^(30,31)^ Besides, salivary phenylalanine and leucine decrease in patients with oral squamous cell carcinomas (OSCC) compared to healthy controls, and this correlates with the stage of OSCC.^(32)^ Lactic acid increases and valine decreases significantly in saliva of patients with OSCC compared to control groups.^(33)^ In carcinogenesis models of rodents, little information is available about salivary metabolites because it is difficulty to collect salivary samples from rodents (usually a very small amount). Thus, metabolome changes in the blood and cancer tissue are reported, and significant changes among several dozen metabolites between carcinogenic and control mice are reported.^(23,24,34)^ In the present study, serum metabolites did not change between Fx-treated and control mice (Table 2). Further investigation is needed to elucidate the correlation among tumor, salivary and serum metabolites in carcinogenic mouse models by Fx administration.

In summary, Fx significantly suppressed the tumor incidence (development) and Cyclin D1 expression in the xenograft mice along with increasing a tumor metabolite predictor, glycine. Our results imply the possibility of tumor metabolites as a prediction marker of tumorigenicity derived from colorectal cancer stem cells in mice. Further studies are needed to clarify the anti-carcinogenic properties of Fx and its impact on tumor metabolites in human.

## Figures and Tables

**Table 1:** Tissue gram weight in xenograft mice with fucoxanthin treatment

Treatment^a^	Liver	Kidney		Spleen	Tumor
		Right	Left		
Fx	1.11 ± 0.04^b,^*	0.20 ± 0.01*	0.20 ± 0.01	0.09 ± 0.01	0.05 ± 0.02
Control	1.23 ± 0.04	0.22 ± 0.01	0.21 ± 0.01	0.10 ± 0.01	0.03 ± 0.01

^a^The xenograft mice with fuxoxanthin (Fx) water or water only (control) treatment. ^b^Mean ± SE (*n* = 10). *p<0.05 (Wilcoxon rank sum test, vs control group).

**Table 2:** Metabolite profile in tumor, saliva and serum of xenograft mice with fucoxanthin treatment

Peak no^a^	Compound	pmol metabolite/mg tumor protein content			pmol metabolite/100 µl saliva			pmol metabolite/100 µl serum	
		Control^b^	Fx^c^		Control	Fx		Control	Fx
	Amino acid								
2	Isoleucine	1.5 ± 0.7^d^	0.1 ± 0.1		ND	ND		8.1 ± 1.7	8.2 ± 1.3
3	Proline	0.5 ± 0.3	ND		ND	ND		0.7 ± 0.2	1.2 ± 0.5
4	Glycine	13.9 ± 2.8	6.7 ± 1.1 *		121.7 ± 29.8	272.2 ± 36.5 *		31.6 ± 10.5	42.3 ± 6.3
8	Alanine	1.2 ± 0.5	0.2 ± 0.2		7.9 ± 7.9	41.8 ± 10.4		6.6 ± 1.2	7.6 ± 1.3
9	Serine	0.1 ± 0.1	ND		9.4 ± 9.4	51.9 ± 12.7		1.6 ± 0.3	2.2 ± 0.5
10	Threonine	0.3 ± 0.3	ND		14.4 ± 14.4	48.5 ± 16.6		13.1 ± 5.1	4.1 ± 0.7
12	Aspartic acid	0.1 ± 0.1	ND		41.4 ± 3.7	103.6 ± 22.8		4.3 ± 2.2	2.5 ± 1.1
14	Glutamic acid	ND	ND		17.9 ± 2.4	51.6 ± 10.9 *		4.8 ± 2.8	0.5 ± 0.4
	Dicarboxylic acid (TCA cycle)								
5	Succinic acid	5.0 ± 0.6	3.9 ± 0.4		25.7 ± 3.0	52.1 ± 4.3 *		7.9 ± 2.2	7.2 ± 1.9
6	Fumaric acid	0.4 ± 0.3	ND		4.8 ± 4.8	14.0 ± 5.3		3.0 ± 0.4	2.3 ± 0.9
11	Malic acid	3.1 ± 0.7	1.5 ± 0.2		82.2 ± 15.8	105.7 ± 15.5		28.9 ± 3.2	17.4 ± 5.8
	Carboxylic acid - others								
1	Butyric acid	13.2 ± 3.2	21.0 ± 8.9		144.3 ± 35.8	264.3 ± 45.4		12.3 ± 1.2	14.9 ± 1.0
7	Pelargonic acid	1.6 ± 0.2	1.1 ± 0.7		37.5 ± 6.5	29.0 ± 7.5		2.8 ± 0.2	3.2 ± 0.3
13	γ-Aminobutyric acid	4.7 ± 1.8	2.1 ± 0.7		ND	ND		6.9 ± 2.8	9.6 ± 1.2

^a^The number of peaks shown in Fig. 5. ^b,c^The xenograft mice with fuxoxanthin (Fx) water or water only (control) treatment. ^d^Mean ± SE (*n* = 4). *p<0.05 (Wilcoxon rank sum test, vs control group).

## Abbreviations

CCSC: colorectal cancer stem cell
CD44s: CD44 standard form
CD44v: CD44 variant forms
Csps: colonospheres
DMEM: Dulbecco’s modified Eagle’s medium
DW: distilled water
Fx: fucoxanthin
PCs: parent cells
SCM: stem cell medium
